# The Surgery of the Heart and Lungs

**Published:** 1904-09

**Authors:** 


The Surgery of the Heart and Lungs. By B. Merrill Rickets, Ph. B., M.D. The Grafton Press: New York, 1904.

In this work, the author seeks to systematize the knowledge of the surgery of the thoracic viscera. The book treats of the surgical conditions found in them and with clinical and experimental research in man and the lower animals with respect to operative procedures. All of these conditions are elaborately set forth. The references to literature are exceedingly full, and illustrations unstinted. The book is unique, and will command the immediate attention of those interested in this province of medicine.

